# Biomechanical effects of lumbar fusion surgery on adjacent segments using musculoskeletal models of the intact, degenerated and fused spine

**DOI:** 10.1038/s41598-021-97288-2

**Published:** 2021-09-09

**Authors:** Mahdi Ebrahimkhani, Navid Arjmand, Aboulfazl Shirazi-Adl

**Affiliations:** 1 Department of Mechanical Engineering, Sharif University of Technology, 11155-9567 Tehran, Iran; 2 Division of Applied Mechanics, Department of Mechanical Engineering, Polytechnique, Montréal, QC Canada

**Keywords:** Biomedical engineering, Mechanical engineering

## Abstract

Adjacent segment disorders are prevalent in patients following a spinal fusion surgery. Postoperative alterations in the adjacent segment biomechanics play a role in the etiology of these conditions. While experimental approaches fail to directly quantify spinal loads, previous modeling studies have numerous shortcomings when simulating the complex structures of the spine and the pre/postoperative mechanobiology of the patient. The biomechanical effects of the L4–L5 fusion surgery on muscle forces and adjacent segment kinetics (compression, shear, and moment) were investigated using a validated musculoskeletal model. The model was driven by in vivo kinematics for both preoperative (intact or severely degenerated L4–L5) and postoperative conditions while accounting for muscle atrophies. Results indicated marked changes in the kinetics of adjacent L3–L4 and L5–S1 segments (e.g., by up to 115% and 73% in shear loads and passive moments, respectively) that depended on the preoperative L4–L5 disc condition, postoperative lumbopelvic kinematics and, to a lesser extent, postoperative changes in the L4–L5 segmental lordosis and muscle injuries. Upper adjacent segment was more affected post-fusion than the lower one. While these findings identify risk factors for adjacent segment disorders, they indicate that surgical and postoperative rehabilitation interventions should focus on the preservation/restoration of patient’s normal segmental kinematics.

## Introduction

Adjacent segment degeneration (ASDeg) and adjacent segment disease (ASDis) are commonly detected conditions, respectively without and with clinical symptoms, following spinal fusion surgeries^1^. While up to 43% of patients may develop postoperative ASDis, the prevalence of ASDeg is much greater (~ 84%)^2^. Some postoperative conditions are the likely consequences of a pre-existing or a natural progress of degeneration. In addition, the causative mechanobiology role of the altered biomechanics following a fusion surgery has been indicated. This includes postoperative alterations in the mobility of adjacent segments, disruptions in their anatomy, and iatrogenic intraoperative injuries to paraspinal muscles which altogether may change the spinal alignment and loadings thereby initiating/accelerating adjacent segment disorders (ASDs)^3–7^.

In vitro, in vivo and in silico biomechanical studies corroborate such postoperative alterations in the spine kinematics and kinetics^8–10^. Image-based in vivo studies can quantify only the postoperative alterations in vertebral kinematics/motions^11^. In vitro studies also remain limited by the assumed idealized loading/boundary conditions^12^. In silico modeling investigations, however and while simulating changes in kinematics, offer an improved insight into postoperative alterations in the kinetics of adjacent discs. Passive finite element (FE) models driven by pure moments with/without follower loads (i.e., forces that follow deformation of the spine)^13–18^ or driven by image-based displacements^19^ as well as musculoskeletal (MS) models with idealized passive joints^20–25^ have been used to investigate the adjacent segments effects. While, force-controlled passive FE models fail to account for the crucial role of muscles^19,26^, displacement-controlled models remain sensitive to measurement errors in vertebral translations^27^.

Realistic MS models are appropriate tools to quantify spine loads under muscle exertions and in vivo activities^28–30^. Previous MS models have investigated the effects of fusion surgeries on adjacent segments by taking into account the altered postoperative kinematics^20–22,24,25^, posture (local/global lumbar lordosis, sacral slope and sagittal vertical axis)^20–22^ and/or iatrogenic intraoperative muscle injuries^23^. Intervertebral joints are however idealized; spherical joints with fixed centers of rotation^20,22,24^ and beam-like elements (stiffness matrix or bushing) with linear properties^21,23^ have been used. Nonlinear behavior of spinal joints^31–34^, joint stiffening under large compressive loads^34–36^, incorporation of translational degrees of freedom^37,38^ and load-dependent location of centers of rotation^32,39^ have generally been overlooked. In addition, by ignoring changes in intervertebral angles from the unloaded posture (initial supine/prone conditions) to the upright standing posture under gravity loads, pre-existing segmental passive moments at the latter posture are often overlooked^20,22^.

Proper simulation of the fusion surgery effects is also crucial. For instance, iatrogenic muscular injuries have been represented by a complete, instead of a partial, removal of the contractile cross-sectional area (CSA) of muscle fascicles in the surgically treated segments^23^. Moreover and instead of in vivo images, postoperative alterations in the vertebral rotations are taken based on in vitro data^21,22^, assumed values^20,24^ or a force-dependent kinematics method^25^. The likely postoperative alterations in the lumbopelvic rhythm (LPR) have neither been considered. Another important concern, often overlooked^20–25^, is the consideration of the preoperative state with a severe disc degeneration^40–43^. Previous studies have generally considered an intact (healthy) preoperative condition, i.e., patients with intact motion at the injured segment (cases with a trauma/tumor/low-grade degenerative spondylolisthesis). Consequences of preoperative disc degeneration in patients with high-grade degeneration and substantial loss of motion/disc height at the injured segment on postoperative outcomes, hence, remains to be investigated.

We aim here to analyze the distinct and combined effects of a L4–L5 fusion surgery on the overall adjacent segments kinetics by incorporating various pre- and postoperative alterations in posture, kinematics, and muscle CSAs. A number of commonly-performed daily activities are simulated by our anatomically-detailed validated nonlinear MS model^28,44,45^. Apart from simulating intact and degenerated preoperative conditions, alterations in the postoperative kinematics (e.g., individual vertebral kinematics, pelvic kinematics, and lumbopelvic rhythm), spinopelvic configuration (e.g., lumbar lordosis and sacral slope) as well as iatrogenic intraoperative muscle injuries are considered all based on available in vivo data. We hypothesize that post-fusion biomechanical changes affect the internal loading of the adjacent segments with the potential to initiate/accelerate ASDs. It is further hypothesized that the extent of such postoperative alterations depends on the preoperative conditions (intact versus degenerated), task (upright versus moderate/large flexion), and adjacent segment (cranial versus caudal).

## Methods

Two distinct models are considered for the preoperative and one for the postoperative simulations. They represent patients with: (1) a preoperative L4–L5 segment showing near-normal (intact) motion in cases with a trauma/tumor/low-grade degenerative spondylolisthesis (“Intact MS model”), (2) a preoperative high-grade L4–L5 degenerated segment with substantial loss of motions and disc height (“Preoperative degenerated MS model”), and (3) a postoperative fused L4–L5 segment (“Fused postoperative MS models”). Intact, preoperative degenerated and postoperative fused models are described below.

### Intact MS model

**Figure 1:**
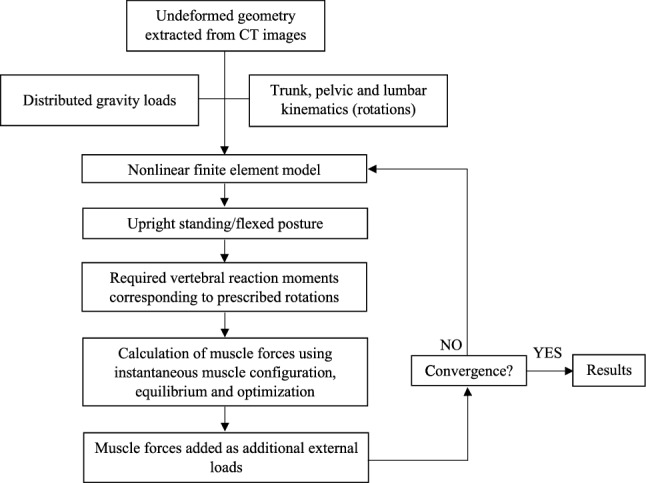
The workflow of the musculoskeletal (MS) model to calculate unknown muscle forces and spinal loads.

**Figure 2:**
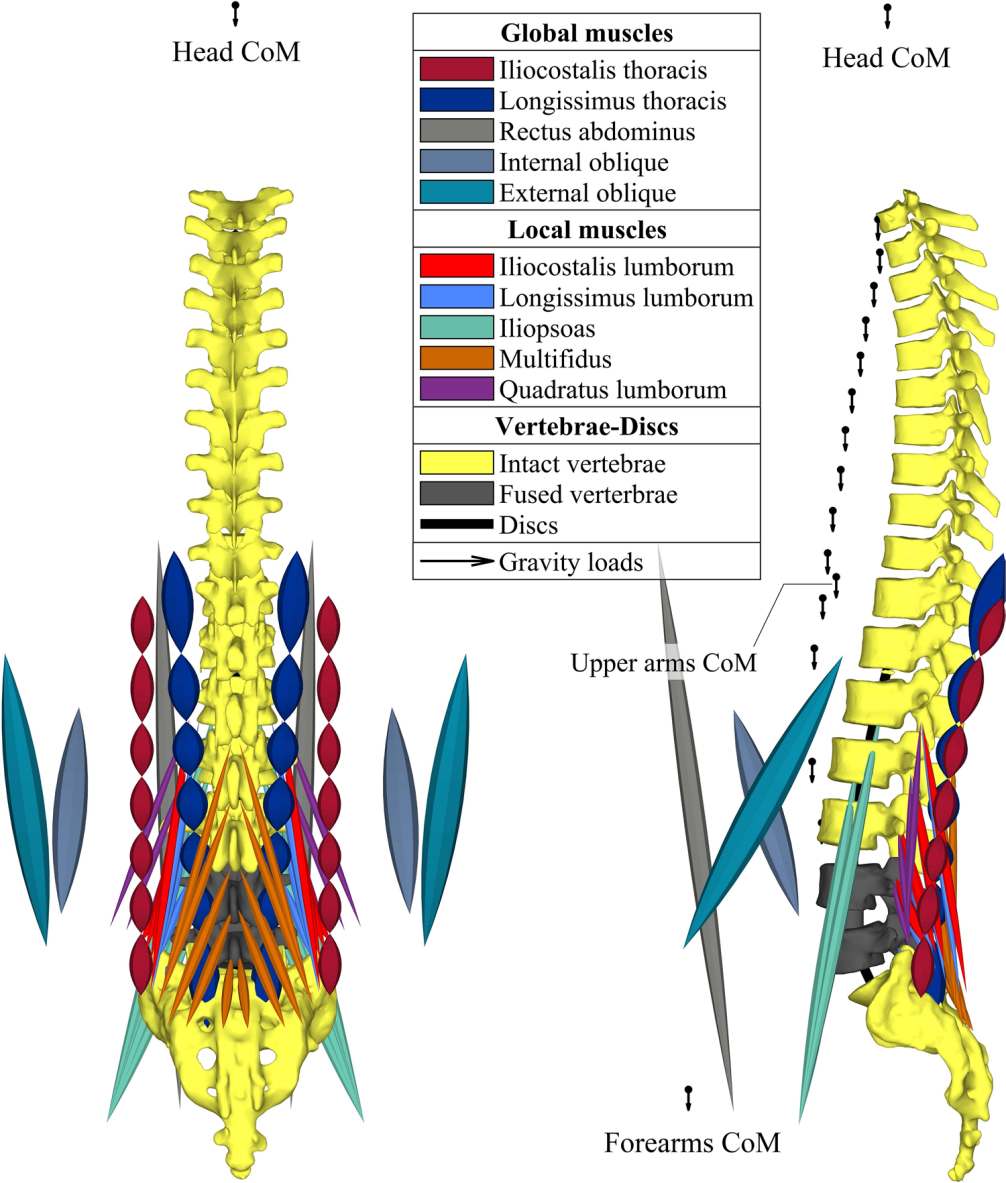
A schematic of the musculoskeletal (MS) model with the trunk (10 global and 46 local) musculature in (left) the frontal (back view) and (right) sagittal (left view) planes. The L4 and L5 vertebrae that are interconnected by the L4-L5 disc in the intact model are instead fused in the post-fusion model (highlighted here in dark grey).

Our extensively-validated nonlinear MS model^28,44–47^ evaluates forces in trunk muscles and spinal joints during in vivo activities using a kinematics-driven optimization algorithm (Figs. 1 and 2). The pelvis, T1–T12 thorax and lumbar vertebrae are rigid. T12–L1 through L5–S1 discs are simulated by 3-node nonlinear shear-deformable beams located 4 mm posterior to the disc centers to account for the shift in the disc center of rotation^32,48,49^. The trunk weight was partitioned among upper arms, forearms, hands, head, and T1–L5 segments and applied via rigid elements at their respective centers of mass^26,44,45,50^. In total, 56 trunk muscle fascicles are incorporated while considering curved lines of action of back global muscles (i.e., their wrapping around and contact forces at bony vertebrae)^46^.

For each simulated task in upright and flexed postures (see “Simulated tasks”), the flexion movement of thorax (T) and pelvis (P) are prescribed into the model based on in vivo measurements^44,45^. The lumbar flexion movement (L = T – P) is subsequently partitioned between the T12–L1 through L5–S1 segments by 11.5%, 15%, 14%, 18%, 21.5% and 20% respectively, based on in vivo studies^45,51,52^. To determine muscle forces, a multi-level optimization algorithm minimizing the sum of cubed muscle stresses is used. In this procedure, the reaction moment of each vertebra is balanced by the corresponding muscles attached to that vertebra. Calculated muscle forces are then fed back into the nonlinear MS model as external loads and muscle forces are recalculated. This iterative approach is continued till convergence is reached, i.e., almost no change in the predicted muscle forces between two successive iterations (Fig. 1). The MS model has been validated in terms of predicted muscle forces and disc compressive loads by comparisons with the measured muscle EMG and L4–L5 intradiscal pressure (IDP) values, respectively, under various tasks in upright/flexed postures in static/dynamic conditions^28,44,45,53,54^.

### Preoperative degenerated MS model

**Figure 3:**
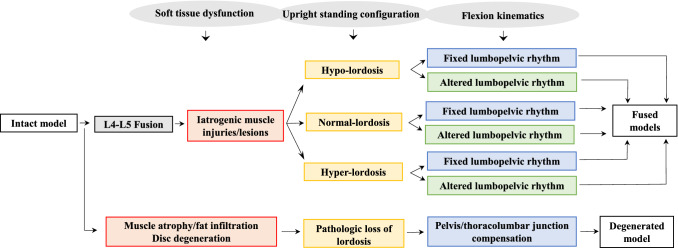
Flowchart of the conditions considered to simulate preoperative (intact and degenerated) as well as fused models.

**Figure 4:**
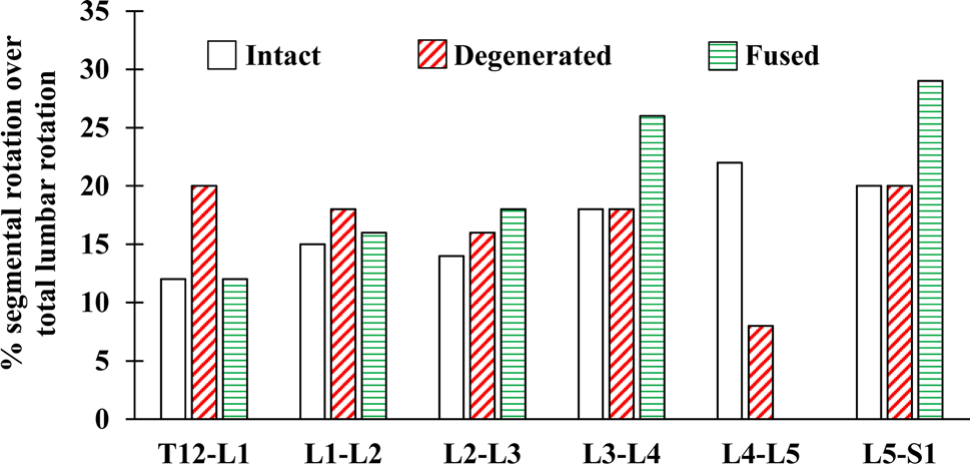
Relative contribution of different lumbar segments to the total lumbar flexion in the intact, degenerated, and fused models.

This simulates a severely degenerated L4–L5 disc with a height loss in the preoperative state (Fig. 3). Muscle atrophy/sarcopenia/fat infiltration are incorporated in the model by reducing their PCSAs^55^ based on our preoperative measurements (on 6 patients) using MR images (14% for MF and 11% for ES in all fascicles which cross over L4–L5 level)^56,57^. Disc degeneration is modelled at the L4–L5 segment by reducing the disc height by two-third according to Pfirrmann classification^58^. Based on in vivo data collected on 44 low back pain patients, LPR decreases (the contribution of pelvis to forward trunk flexion increases) as compared to healthy individuals^59,60^. Moreover, MR images (of 259 patients)^61^ indicate that the loss of motion at the degenerated (i.e., stiffened) L4–L5 segment is compensated by the hypermobility of motion segments at the thoracolumbar junction rather than the cranial or caudal adjacent segments. Therefore, in the preoperative degenerated model, LPR is dropped by 20% and the lumbar vertebral rotations are revised (compensating reduced motion at the L4–L5 by larger angles at the T12–L1 and L1–L2 levels; i.e., the contribution of L4–L5 over total lumbar rotation was reduced from 21.5% in the intact model to 8% and the contribution of T12–L1 and L1–L2 increased from 11.5% and 15% in the intact model to 19.5% and 18%, respectively) (Fig. 4). Note that the reduced rotation at the L4–L5 segment is a direct consequence of the higher stiffness (implemented in the model by reducing the disc height) at the severely narrowed degenerated disc.

### Fused postoperative MS models

Fusion simulations are solely considered for the intact MS model. This is because similar postoperative models are expected irrespective of the preoperative conditions (see “Limitations” section). Fused model is actually the intact model modified by rigidly connecting the L4 and L5 vertebrae (Fig. 2). The postoperative geometry of the spine, prescribed kinematics of vertebrae/pelvis, and physiological cross-sectional areas of injured muscles are subsequently modified based on available in vivo data as described below (also summarized in Fig. 3).

#### Upright standing spinal posture

Simulated postoperative alterations in the upright standing posture includes changes in the segmental (local) L4–L5 lordosis, lumbar (global) lordosis, and sacral slope. From a neuro-muscular perspective, in order to keep the balance, the body assumes a posture with minimum muscular energy requirement in neutral upright standing^62,63^. The fusion technique [posterior lumbar fusion (PLF), posterior lumbar interbody fusion (PLIF), anterior lumbar interbody fusion (ALIF) or transforaminal lumbar interbody fusion (TLIF)]^64,65^, the shape of intervertebral cage (rectangular or wedge-shaped)^66,67^, and the surgical set-up (intraoperative patient position)^68,69^ influence the postoperative posture. This adapted postoperative standing posture can be estimated through an optimization procedure^63^. Three postoperative L4–L5 lordosis angles are considered in the current MS model; normal-lordosis (no changes between pre- and postoperative L4–L5 lordoses), hyper-lordosis (increased lordosis), and hypo-lordosis (reduced lordosis)^21,70^. Postoperative changes in the lordosis of the fused segment (L4–L5) in cases of hyper- and hypo-lordoses are taken according to an in vivo study (with 42 patients)^66^. Rotations at the remaining intact segments including the sacrum are subsequently calculated via an optimization procedure that minimizes the sum of joint reaction moments and thus muscle forces in the upright standing posture under gravity loading^47,54^. These optimal rotations from the unloaded supine state to the upright standing under gravity loads are given in Supplementary Table S1 for intact, degenerated and fused models for the three normal, hyper- and hypo-lordosis configurations. Postures are depicted in Supplementary Figure S1.

#### Segmental kinematics in flexion tasks

Based on in vivo observations, two scenarios are adopted here; once it is assumed that the postoperative lumbopelvic rhythm (i.e., ratio of total lumbar over pelvis rotations, LPR) remains unchanged and thus the eliminated L4–L5 motion after fusion is compensated by the remaining individual lumbar segments (L1–L5) (an approach similar to that in previous MS studies^22,25^) (Supplementary Table S2). In order to do so, the individual lumbar motion rhythms (i.e., ratio of segmental over total lumbar rotations) are modified based on upright x-ray measurements of motion rhythms in 36 patients before and after the fusion surgery^71^, i.e., the L4–L5 rotation is distributed among L1–L2 to L5–S1 by 3%, 19%, 38%, 0% and 40%, respectively (Fig. 4). According to another in vivo study on 5 patients^72^, LPR alters after the spinal fusion surgery in some patients (contribution of pelvis increases in flexion). Therefore, in the second scenario, the eliminated L4–L5 rotation after fusion is compensated by the pelvis alone, i.e., pelvis rotations were increased by 2.8°, 6°, 9.3° and 11.1° in trunk flexion angles of 20°, 40°, 60° and 80°, respectively (Supplementary Table S2). The rotations between other intact lumbar segments are again distributed according to the postoperative fused rhythms (Fig. 4 and Supplementary Table S2). To reach the final flexed postures, the vertebral (T12 through S1) rotations for different flexed postures (Supplementary Table S2) are applied to the upright standing posture (Supplementary Table S1).

#### Muscle iatrogenic injuries

Iatrogenic intraoperative injuries in back muscles are simulated according to our previous measurement (on 6 patients) and modeling studies^56,57^; the physiological cross-sectional areas (PCSAs) of multifidus (MF) and erector spinae (ES) fascicles crossing over the L4–L5 level are reduced in postoperative models by 26% and 11%, respectively. To investigate the effect of muscle damages alone on adjacent segment kinetics in flexion tasks, the largest (80°) trunk flexion task (section Simulated tasks) was re-simulated in the intact state by only implementing muscle damages (considering identical kinematics).

### Simulated tasks

Some commonly-performed daily activities including the neutral upright standing posture and forward flexion of 20, 40, 60, and 80° with arms in the gravity direction are simulated by the intact, preoperative degenerated, and various postoperative fused MS models. These tasks are also chosen due to the availability of the required pre- and postoperative kinematics to drive simulations^45,61,71^.

### Model outputs

Following the hypothesis that ASDs are likely a consequence of alterations in the loading patterns, the following outputs are calculated for the intact, degenerated, and fused conditions: segmental local compression/shear loads and passive joint moments at the adjacent discs mid-height planes (i.e., L3–L4 and L5–S1) as well as the muscle forces. A substantial change (assumed here to be > 25%), as compared to the preoperative intact or degenerated states, in these parameters highlights an increase in the risk to initiate/accelerate postoperative ASDs.

## Results

### Intact model

The intact model has been validated elsewhere^28,44,45,53,54^.

### Preoperative degenerated model

**Figure 5:**
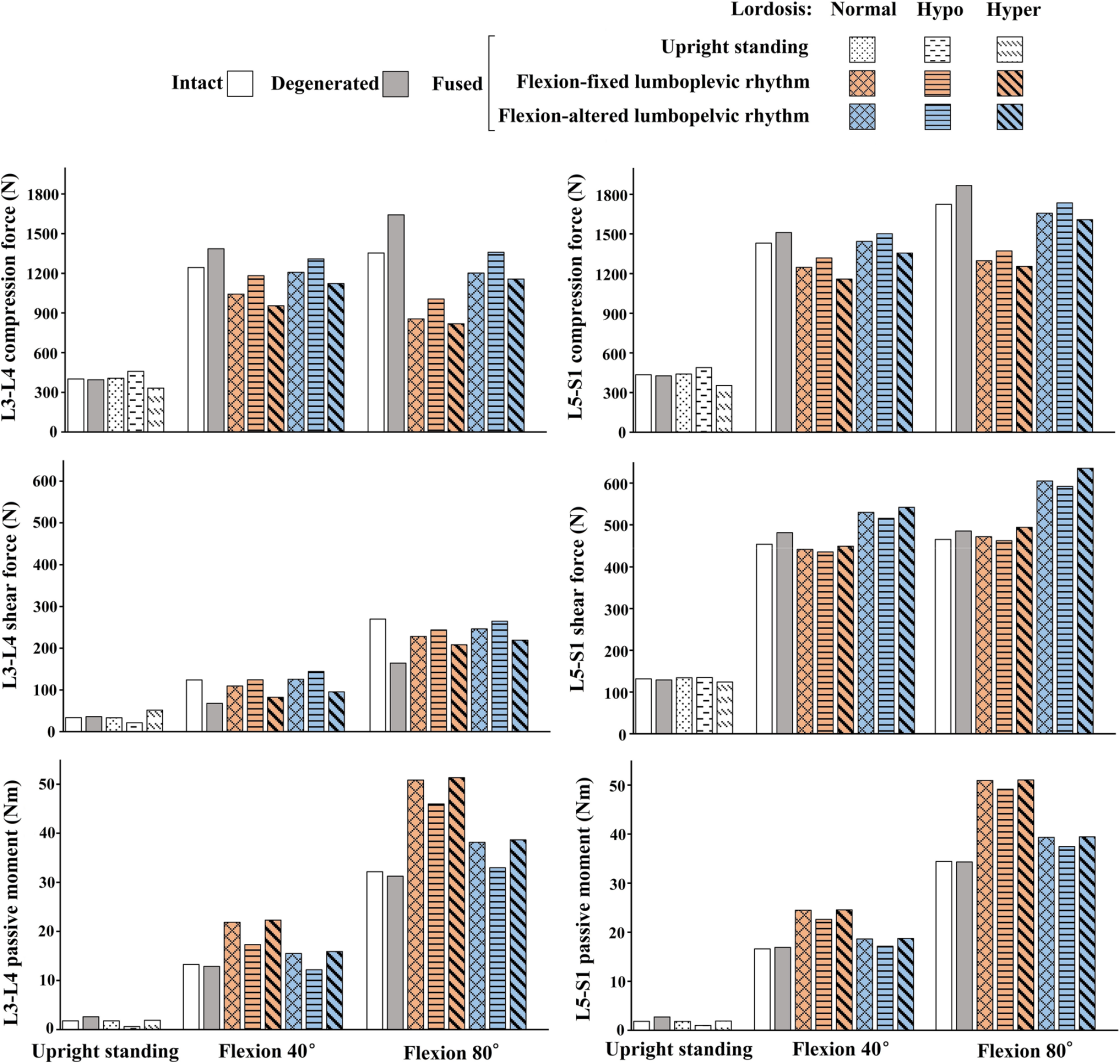
Compression force (N) (top), shear force (N) (middle) and passive moment (Nm) (bottom) at the upper (L3-L4) (left) and lower (L5-S1) (right) adjacent disc mid-planes for the preoperative intact and degenerated as well as postoperative fused states in the upright and two flexed postures (40 and 80°). Results for all simulated flexion tasks are given in Supplementary Table S3.

In the upright standing posture, predictions were found close enough to those in the intact model (changes < 10%, Table 1 and Fig. 5). In flexion tasks, the increase in the T12–L2 flexion angles (Fig. 4) and pelvic flexion (Supplementary Table S2) significantly reduced global and increased local muscle forces (Table 2). Consequently, compression forces increased by up to 21% and shear forces decreased by up to 48% at the upper adjacent segment (Table 1). All other alterations remained < 10%.

**Table 1:**
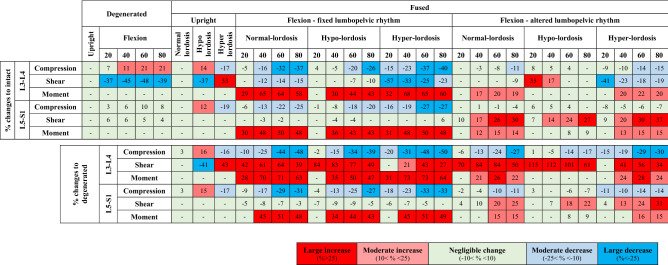
Changes (%) in adjacent segment compression and shear loads as well as passive moment in fused/degenerated states relative to intact state (upper section) and in fused states relative to degenerated state (lower section) in upright and flexed postures. Magnitude of changes is depicted by five color-coded levels (see the legend below). Small net changes (e.g., less than 2 Nm for passive moments or 10 N for compression and shear forces) are marked with (-) irrespective of their relative changes.

**Table 2:**
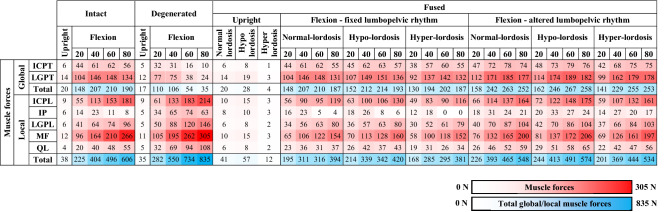
Unilateral forces (N) in local muscles (ICPL: iliocostalis lumborum pars lumborum, LGPL: longissimus thoracis pars lumborum, IP: iliopsoas, MF: multifidus; QL: quadratus lumborum) and global muscles (ICPT: iliocostalis lumborum pars thoracis, LGPT: longissimus thoracis pars thoracis) as well as the sum of local and global muscle forces. No forces in abdominal muscles (IO: internal oblique; EO: external oblique; RA: rectus abdominus) were computed in all simulated tasks. Please note the legends at the bottom for the color highlights.

### Postoperative models

Postoperative kinetics of adjacent segments altered not only with the spinal lordosis, LPR, segmental kinematics and muscle injuries but also with the level of adjacent segment, preoperative L4–L5 disc condition, and simulated task (Table 1, Fig. 5, Supplementary Table S3). In general, alterations in LPR and muscle injuries had, respectively, the greatest and least effects on adjacent segment kinetics. Larger postoperative changes generally occurred at the upper adjacent segment and in flexion tasks especially when the postoperative LPR remained unchanged with respect to preoperative conditions (Table 1 and Fig. 5). More details on task-specific findings are provided below.

#### Upright standing

Compared to preoperative intact and degenerated states, the postoperative hypo-lordosis posture increased external flexion moments, local/global muscle forces (Table 2), and compression forces (though by < 16%) at both adjacent segments (Table 1). Shear force decreased at the upper segment by up to 41% (Table 1). In contrast, the hyper-lordosis condition decreased external flexion moments, all muscle forces (Table 2) and consequently adjacent segment compression forces by up to 19% as compared to preoperative states (Table 1). In this configuration, the shear force increased at the upper segment by up to 53%. Irrespective of the hypo or hyper-lordosis postures, passive moments at both adjacent segments and shear force at the lower segment were only slightly affected (< 10%) when compared to those in both preoperative states (Table 1). Moreover, the postoperative normal-lordosis configuration yielded results almost the same as those in the preoperative intact and degenerated states with alterations in all cases < 10% (Table 1). Muscle injuries had slight effects in local muscle forces, compression forces, shear forces and passive moments at adjacent segments (all < 10%) (Tables 1,2).

#### Flexed postures

With the fixed postoperative LPR, larger flexion angles at the adjacent segments significantly increased their passive moments by up to 73% (Table 1 and Fig. 5). This reduced local muscle forces and adjacent segment compression loads when comparing to preoperative flexed states (Tables 1, 2). At the upper adjacent segment, shear forces generally decreased with respect to the intact state but significantly increased when compared to the degenerated state (Table 1). With the altered postoperative LPR compared to the fixed LPR, adjacent segment effects were generally less pronounced with the exception of the upper adjacent segment shear loads which increased by up to 115% when compared to the degenerated state (Table 1). The effect of muscle damages alone (with no postoperative alteration in kinematics) on adjacent segment kinetics was found < 3% in 80° trunk flexion task.

## Discussion

Biomechanical effects of the L4–L5 fusion surgery on adjacent segment kinetics were investigated while considering two distinct preoperative L4–L5 disc conditions. A validated MS spine model driven by in vivo data on pre- and postoperative T12–S1 kinematics was employed while also simulating alterations in spinal lordosis and muscle injuries. In corroboration of our hypotheses, marked postoperative alterations were predicted in adjacent segment kinetics that depended on the preoperative L4–L5 disc condition, postoperative lumbopelvic kinematics and, to a lesser extent, the postoperative changes in the L4–L5 segmental lordosis and intraoperative muscle injuries. Moreover, upper (L3–L4) and lower (L5–S1) adjacent segment kinetics were affected post-fusion to different degrees.

### Lower (L5–S1) adjacent segment

Fixed LPR postoperatively generally increased passive moments by up to 51% but decreased compression forces by up to 33% compared to preoperative cases (Table 1). These changes, that were due primarily to the increased segmental flexion angles at the lower adjacent level, may increase tensile stresses-strains in disc annulus matrix and fibers at adjacent levels^30,49,73^. In the model with an altered postoperative LPR, foregoing alterations nearly disappeared despite an increase in the shear force by up to 37% under larger trunk flexion angles. All other postoperative alterations in the upright and flexed postures remained < 25%. In agreement with another model study^21^, in flexion tasks and at the lower adjacent level, hyper- and hypo-lordosis configurations produced the largest and smallest shear loads, respectively. The alterations in local shear forces depend on the alterations in the disc inclination and the forces in local/global muscles (Table 2).

### Upper (L3–L4) adjacent segment

Alterations in the postoperative kinetics were generally more pronounced in the upper adjacent segment than the lower one (Table 1). This finding is in agreement with clinical observations showing greater prevalence of ASDs at the upper adjacent segment^74–79^. Even in the upright posture, large postoperative alterations in the L3–L4 shear load by up to 53% and 43% in the hyper-lordosis fused model with, respectively, intact and degenerated L4-L5 discs were predicted (Table 1). In agreement with other model studies^24,25^, fixed LPR postoperatively was found to generally increase passive moments and lower compression forces with respect to preoperative cases (Table 1). Moreover, L3–L4 shear load in flexed postures significantly increased by up to 84% as compared to the degenerated condition. In corroboration, in vivo animal studies and in vitro tests have shown that the shear^80,81^ and hyperflexion^82^ loads increase the risk of disc degeneration. It therefore appears that in these patients, the upper segment ASDs are likely associated with foregoing changes in segmental biomechanics. In patients with an altered postoperative LPR, however, these alterations were subdued and generally limited to an increase in the L3–L4 shear load by up to 115% only in those with the severely degenerated preoperative state (Table 1). Finally, due to alterations in disc inclination and muscle forces (Table 2), hyper- and hypo-lordosis configurations in flexion tasks produced the smallest and largest shear loads, respectively^21^.

### Surgically altered segmental lordosis

Alterations in the L4–L5 segmental lordosis perturbed postoperative postures. For instance, the hypo-lordosis configuration caused an additional forward bending of the thorax, increased sagittal vertical axis (SVA) and retroversion of pelvis, i.e., a smaller sacral slope (Supplementary Figure S1). In contrast, the hyper-lordosis configuration resulted in the backward bending of the thorax, decreased SVA and anteversion of pelvis, i.e., a larger sacral slope (Supplementary Figure S1) thereby reducing all muscle forces (Table 2) and adjacent segment compression forces. In accordance with our findings, numerous clinical studies recommend correcting preoperative lumbopelvic abnormalities and restore the segmental lordosis during the surgery^83–86^.

### Traditional versus minimally invasive surgeries

The minimally invasive fusion surgery, as compared to the conventional open technique, considerably reduces the risk of iatrogenic intraoperative muscle injuries^87^ and likely the ASDs^88^. Our predictions (Table 2), in agreement with a previous modeling study^57^, however, demonstrated that intraoperative muscle injuries affected primarily the load sharing among muscles with minimal direct alterations on adjacent segment kinetics. In agreement, several clinical studies have reported similar ASD prevalence among patients undergoing the minimally invasive fusion surgery or the conventional open techniques^78,89,90^. In contrast, a model study reported large changes in adjacent segment kinetics following the removal of entire muscle fascicles at treated levels^23^. In the standing posture, for instance, increases of 78% and 82% in upper adjacent segment compression and shear forces, respectively, were reported^23^.

### Passive FE models

Passive FE models use idealized follower load/moment loading scenarios to mimic in vivo loading conditions^13,14,17,18,91–94^. Such a purely compressive follower load influences spinal kinematics/kinetics and overlooks shear loads in adjacent segments^26^. Our results, however, show important post-fusion changes in adjacent segment shear loads (Table 1). Furthermore, these models generally use a force control protocol with identical loads in pre- and post-operative conditions. Consequently, they estimate, unlike our findings here, postoperative increases in adjacent segment IDPs^13,14,17,18,92–94^.

### Limitations

Available image-based in vivo kinematics data for intact, degenerated and fused conditions are dispersed and limited^11,42^. Fusion simulations were not performed on the preoperative degenerated models. It is expected that the influence of the preoperative disc degeneration on spinal kinematics diminishes after the surgery by restoring the disc height with an interbody fusion device. Our MS model employs an optimization method to estimate muscle forces. It is unknown whether the central nervous system controls muscle forces in a compatible manner especially in patients. Moreover, the model used in this study is generic and neglects patient-specific parameters such as the spinal sagittal alignment, passive properties, and muscle characteristics. Finally, MS models have intrinsic limitations in not predicting internal stress/strain in ligaments, facet joints, vertebrae, and disc annulus/fiber networks. The gold standard model to investigate the biomechanical effects of pathological conditions remains the novel coupled MS-FE model of the spine that simultaneously incorporates detailed architecture of muscles and joints^49^.

### Conclusions

The preoperative disc condition, postoperative alterations in segmental lordosis/lumbopelvic rhythm/vertebral movements, and adjacent level position affected the alterations in adjacent segment biomechanics. The primary variable influencing the adjacent segment kinetics when reaching a unique posture was, however, the extent of differences in the lumbar segmental flexion angles between preoperative and postoperative states. Surgical interventions should therefore focus on making minimal changes in movement patterns while postoperative rehabilitation programs should attempt to restore near normal motions.. Any indirect modifications in the global alignment and pelvic flexion angle, for example following a rehabilitation program, that change the lumbar segmental kinematics will inevitably also influence spinal forces. Finally, in vivo kinematics data for intact, degenerated and fused conditions under various activities are needed in patients for more accurate simulations.

## Supplementary Information

Supplementary Information.
