# Supplementary material for: Biomechanical effects of lumbar fusion surgery on adjacent segments using musculoskeletal models of the intact, degenerated and fused spine
Source: Sci Rep. 2021 Sep 9;11:17892. doi: 10.1038/s41598-021-97288-2 (PMC8429534; doi:10.1038/s41598-021-97288-2)
Supplement: Supplementary file 1 — Supplementary Information. [file 41598_2021_97288_MOESM1_ESM.doc]

**Supplementary Table S1** - Prescribed kinematics (thorax, pelvis and lumbar rotations in degrees) onto the undeformed FE model to construct the upright standing posture under gravity based on posture optimization outputs (positive values indicate forward flexion relative to the undeformed state). Surgically induced alterations in L4-L5 lordosis, relative to preoperative supine lordosis, for different fused states are reported. In undeformed (supine) configuration, intervertebral angles are 3.0°, 3.4°, 3.4°, 8.5° and 21.2° at L1-L2 to L5-S1, respectively, all based on CT images of a 65 years-old male cadaver^26^. Some select postures are depicted in Supplementary Figure S1.

|  | **Upright standing** | | | | | | | | |
| --- | --- | --- | --- | --- | --- | --- | --- | --- | --- |
|  | **Intact** |  | **Degenerated** |  | **Fused normal-lordosis** |  | **Fused hypo-lordosis** |  | **Fused hyper-lordosis** |
|  |  |  |  |  |  |  |  |  |  |
| **T12** | 4.9 |  | 5.6 |  | 4.9 |  | 5.5 |  | 2.5 |
| **L1** | 1.0 |  | 1.5 |  | 1.0 |  | 1.7 |  | -1.5 |
| **L2** | -2.9 |  | -2.6 |  | -2.9 |  | -2.0 |  | -5.5 |
| **L3** | -5.8 |  | -5.9 |  | -5.8 |  | -4.5 |  | -8.6 |
| **L4** | -7.6 |  | -7.9 |  | -8.2 |  | -8.2 |  | -8.1 |
| **L5** | -8.8 |  | -8.5 |  |  |  |  |  |  |
| **S1** | -10.0 |  | -10.2 |  | -10.0 |  | -12.0 |  | -6.9 |
| **Alteration in L4-L5 Lordosis after fusion** | - |  | - |  | **-1.1** |  | **-6.1** |  | **4.9** |

**Supplementary Table S2** - Prescribed kinematics (thorax, pelvis and lumbar rotations in degrees) into the FE model in upright standing to arrive to the flexed postures for intact, degenerated and fused conditions in two scenarios (fixed and altered lumbopelvic rhythm, LPR). Segmental contribution in total lumbar flexion for intact, degenerated and fused models are reported in Figure 4. Some select postures are depicted in Supplementary Figure S1.

|  | **Flexion 20°** | | | |  | **Flexion 40°** | | | |  | **Flexion 60°** | | | |  | **Flexion 80°** | | | |
| --- | --- | --- | --- | --- | --- | --- | --- | --- | --- | --- | --- | --- | --- | --- | --- | --- | --- | --- | --- |
|  | **Intact** | **Degenerated** | **Fused LPR-fixed** | **Fused LPR-altered** |  | **Intact** | **Degenerated** | **Fused LPR-fixed** | **Fused LPR-altered** |  | **Intact** | **Degenerated** | **Fused LPR-fixed** | **Fused LPR-altered** |  | **Intact** | **Degenerated** | **Fused LPR-fixed** | **Fused LPR-altered** |
|  |  |  |  |  |  |  |  |  |  |  |  |  |  |  |  |  |  |  |  |
| **T12** | 20.0 | 20.0 | 20.0 | 20.0 |  | 40.0 | 40.0 | 40.0 | 40.0 |  | 60.0 | 60.0 | 60.0 | 60.0 |  | 80.0 | 80.0 | 80.0 | 80.0 |
| **L1** | 18.5 | 17.6 | 18.5 | 18.8 |  | 36.8 | 34.9 | 36.8 | 37.5 |  | 55.0 | 51.9 | 55.0 | 56.1 |  | 74.1 | 71.0 | 74.0 | 75.3 |
| **L2** | 16.5 | 15.4 | 16.4 | 17.2 |  | 32.6 | 30.2 | 32.4 | 34.1 |  | 48.5 | 44.7 | 48.2 | 50.8 |  | 66.3 | 62.4 | 66.0 | 69.0 |
| **L3** | 14.7 | 13.5 | 14.1 | 15.3 |  | 28.7 | 26.1 | 27.4 | 30.1 |  | 42.4 | 38.2 | 40.4 | 44.6 |  | 59.1 | 54.8 | 56.6 | 61.6 |
| **L4** | 12.3 | 11.3 | 10.6 | 12.6 |  | 23.7 | 21.5 | 20.1 | 24.4 |  | 34.6 | 30.9 | 29.1 | 35.7 |  | 49.8 | 46.0 | 43.1 | 51.0 |
| **L5** | 9.5 | 10.3 |  |  |  | 17.7 | 19.5 |  |  |  | 25.3 | 27.7 |  |  |  | 38.6 | 42.2 |  |  |
| **S1** | 6.9 | 7.9 | 6.9 | 9.7 |  | 12.1 | 14.3 | 12.1 | 18.1 |  | 16.7 | 19.6 | 16.7 | 26.0 |  | 28.3 | 32.5 | 28.3 | 39.4 |

**Supplementary Table S3** - Compression force (N), shear force (N) and passive moment (Nm) at upper (L3-L4) and lower (L5-S1) adjacent disc mid-planes for the preoperative (intact and degenerated) as well as postoperative fused states in upright and flexed postures. Please see the legends below the table where color highlights are defined.

|  |  |  |  |  |  |  |  |  |  |  |  |  |  |  |  |  |  |  |  |  |  |  |  |  |  |  |  |  |  |  |  |  |  |  |  |  |  |  |  |  |
| --- | --- | --- | --- | --- | --- | --- | --- | --- | --- | --- | --- | --- | --- | --- | --- | --- | --- | --- | --- | --- | --- | --- | --- | --- | --- | --- | --- | --- | --- | --- | --- | --- | --- | --- | --- | --- | --- | --- | --- | --- |
|  |  | | **Intact** | | | | | **Degenerated** | | | | | **Fused** | | | | | | | | | | | | | | | | | | | | | | | | | | |  |
|  |  |  |  |  |  |  |  |  |  |  |  |  | **Upright** | | | **Flexion - fixed lumbopelvic rhythm** | | | | | | | | | | | | **Flexion - altered lumbopelvic rhythm** | | | | | | | | | | | |  |
|  |  |  | **Upright** | **Flexion** | | | | **Upright** | **Flexion** | | | | **Normal lordosis** | **Hypo lordosis** | **Hyper lordosis** | **Normal-lordosis** | | | | **Hypo-lordosis** | | | | **Hyper-lordosis** | | | | **Normal-lordosis** | | | | **Hypo-lordosis** | | | | **Hyper-lordosis** | | | |  |
|  |  |  |  | **20** | **40** | **60** | **80** |  | **20** | **40** | **60** | **80** |  |  |  | **20** | **40** | **60** | **80** | **20** | **40** | **60** | **80** | **20** | **40** | **60** | **80** | **20** | **40** | **60** | **80** | **20** | **40** | **60** | **80** | **20** | **40** | **60** | **80** |  |
|  | **L3-L4** | **Compression** | 401 | 855 | 1244 | 1340 | 1353 | 395 | 911 | 1385 | 1620 | 1642 | 406 | 458 | 332 | 816 | 1043 | 915 | 855 | 891 | 1182 | 1066 | 1006 | 730 | 955 | 847 | 818 | 858 | 1208 | 1231 | 1202 | 923 | 1309 | 1387 | 1358 | 774 | 1123 | 1156 | 1156 |  |
|  |  | **Shear** | 34 | 51 | 124 | 195 | 270 | 36 | 32 | 68 | 102 | 164 | 33 | 21 | 52 | 46 | 109 | 167 | 228 | 59 | 124 | 181 | 244 | 22 | 82 | 146 | 208 | 54 | 125 | 188 | 246 | 69 | 144 | 205 | 265 | 30 | 96 | 159 | 219 |  |
|  |  | **Moment** | 2 | 7 | 13 | 25 | 32 | 3 | 7 | 13 | 24 | 31 | 2 | 1 | 2 | 9 | 22 | 41 | 51 | 7 | 17 | 36 | 46 | 9 | 22 | 41 | 51 | 8 | 16 | 30 | 38 | 6 | 12 | 25 | 33 | 8 | 16 | 30 | 39 |  |
|  | **L5-S1** | **Compression** | 434 | 1001 | 1430 | 1598 | 1724 | 426 | 1031 | 1510 | 1757 | 1866 | 440 | 489 | 354 | 939 | 1248 | 1244 | 1297 | 990 | 1318 | 1312 | 1371 | 845 | 1159 | 1174 | 1255 | 1009 | 1443 | 1585 | 1656 | 1058 | 1501 | 1657 | 1734 | 917 | 1355 | 1509 | 1607 |  |
|  |  | **Shear** | 132 | 335 | 453 | 451 | 465 | 129 | 354 | 481 | 474 | 485 | 134 | 135 | 124 | 337 | 442 | 440 | 471 | 330 | 435 | 433 | 462 | 334 | 448 | 450 | 494 | 368 | 530 | 570 | 605 | 359 | 515 | 559 | 592 | 367 | 542 | 589 | 636 |  |
|  |  | **Moment** | 2 | 9 | 17 | 28 | 34 | 3 | 9 | 17 | 28 | 34 | 2 | 1 | 2 | 11 | 24 | 42 | 51 | 10 | 23 | 40 | 49 | 11 | 25 | 42 | 51 | 9 | 19 | 32 | 39 | 8 | 17 | 30 | 37 | 9 | 19 | 32 | 39 |  |

**51 Nm**

**636 N**

**1866 N**

**0 Nm**

**0 N**

**0 N**

**Compression**

**Shear**

**Passive moment**

**Supplementary Figure S1:** Spinal posture (vertebral positions) in undeformed, fused upright, and fused 40° flexion tasks (altered lumbopelvic rhythm) under different normal, hypo-, and hyper-lordosis conditions.
